# Options for Engineering Apomixis in Plants

**DOI:** 10.3389/fpls.2022.864987

**Published:** 2022-03-14

**Authors:** Pei Pei Yin, Li Ping Tang, Xian Sheng Zhang, Ying Hua Su

**Affiliations:** State Key Laboratory of Crop Biology, College of Life Sciences, Shandong Agricultural University, Tai' an, China

**Keywords:** apomixis, apomeiosis, parthenogenesis, somatic embryogenesis, plant breeding

## Abstract

In plants, embryogenesis and reproduction are not strictly dependent on fertilization. Several species can produce embryos in seeds asexually, a process known as apomixis. Apomixis is defined as clonal asexual reproduction through seeds, whereby the progeny is identical to the maternal genotype, and provides valuable opportunities for developing superior cultivars, as its induction in agricultural crops can facilitate the development and maintenance of elite hybrid genotypes. In this review, we summarize the current understanding of apomixis and highlight the successful introduction of apomixis methods into sexual crops. In addition, we discuss several genes whose overexpression can induce somatic embryogenesis as candidate genes to induce parthenogenesis, a unique reproductive method of gametophytic apomixis. We also summarize three schemes to achieve engineered apomixis, which will offer more opportunities for the realization of apomictic reproduction.

## Introduction

Reproduction is a fundamental, essential process in plant biology and has great practical significance as much of the food supply is seed-based (Fei et al., 2019). Flowering plants follow one of two pathways for propagation through seeds: sexual and asexual reproduction (Vallejo-Marín et al., 2010). During sexual reproduction, two sperm cells combine with the central cell and the egg cell to produce the endosperm and embryo, respectively, during double fertilization (Dresselhaus et al., 2016). A drawback of sexual reproduction is that advantageous traits segregate randomly into different offspring at each generation, often resulting in the loss of advantageous gene combinations (Spillane et al., 2004; Brukhin, 2017). By contrast, asexual reproduction allows the inheritance of the maternal genome without fertilization or genetic recombination (Koltunow and Grossniklaus, 2003). Apomixis produces seeds that are genetically maternal and represents a natural type of asexual reproduction (Noyes, 2008), and it has tremendous potential in agriculture, in particular to preserve heterosis over multiple generations.

Given the significance of apomixis in crop production and breeding, scientists and breeders have worked to introduce apomixis into agronomically important crops, with some well-documented achievements (Hand and Koltunow, 2014; Sailer et al., 2016). In this review, we describe the classification and mechanisms of apomixis, then summarize the research achievements that are paving the way to the introduction of apomixis into sexually reproducing crops. We also highlight the importance of identifying new genes such as *BABY BOOM* (*BBM*) that can lead to somatic embryogenesis, particularly those that may be involved in zygote activation, with the eventual aim of achieving apomixis in agricultural crops.

## Natural Apomixis

Apomixis has two basic types: sporophytic and gametophytic. In sporophytic apomixis, embryos develop from the sporophytic cells of the ovule. Sporophytic apomixis is common in citrus plants, in which diploid ovule cells have an embryogenic cell fate and can form multiple globular embryos *via* mitosis, although their continued development requires the formation of a nutritive endosperm (Hand and Koltunow, 2014). By contrast, in the context of gametophytic apomixis, embryos are derived from the egg cells of diploid or polyploid plants produced by unreduced embryo sacs. Based on the origin of precursor cells that ultimately produce chromosomally unreduced embryo sacs, gametophytic apomixis is subdivided into two types: diplospory and apospory. In diplospory, the precursor is the megaspore mother cell, which undergoes aberrant or suppressed meiosis; in apospory, the precursor is a somatic nucellar cell, resembling the developmental fate of a functional megaspore (Koltunow, 1993; Koltunow and Grossniklaus, 2003; Hand and Koltunow, 2014; Sailer et al., 2016). Gametophytic diplospory has been used in engineered apomixis, whereby diploid or polyploid egg cells are produced by altered or omitted meiosis, referred to as apomeiosis (Hand and Koltunow, 2014). The embryos develop from diploid or polyploid egg cells *via* parthenogenesis without fertilization, while the endosperm of apomictic species develops either without fertilization in autonomous apomicts or following the induction of fertilization in pseudegamous apomicts (Koltunow, 1993; Koltunow and Grossniklaus, 2003).

## Engineering Apomixis

Although apomixis has been documented in over 400 angiosperm species, no major species of agricultural importance are apomictic other than a few fruit crops, such as apple (*Malus domestica*), citrus, and mango (*Mangifera indica*; Wang et al., 2017). To introduce apomixis, composite methods combining apomeiosis with parthenogenesis or genome elimination have been used in rice or Arabidopsis. Genes/alleles (Table 1) revealing phenotypes with potential for engineering apomixis have been identified, as outlined below.

**Table 1:** Genes and their related functions involved in apomeiosis, parthenogenesis, and genome elimination.

Component of apomixis	Gene	Species	Gene product/function	References
Apomeiosis	*DYAD/SWI1*	Arabidopsis	Regulator of meiotic chromosome organization	Ravi et al., 2008; Siddiqi et al., 2000
	*AM1*	Maize	SWI1 ortholog	Pawlowski et al., 2009
	*AtSPO11-1*	Arabidopsis	Topoisomerase-like transesterase	Grelon, 2001
	*AtSPO11-2*	Arabidopsis	AtSPO11-1 paralog	Stacey et al., 2006
	*AtREC8*	Arabidopsis	Cohesin necessary for centromere cohesion and kinetochore orientation	Chelysheva et al., 2005
	*OSD1*	Arabidopsis	Plant-specific protein promoting the transition of meiosis I to meiosis II	D’Erfurth et al., 2009; Cromer et al., 2012
	*TAM/CYCA1;2*	Arabidopsis	Type-A cyclin required for the transition of meiosis I to meiosis II	D’Erfurth et al., 2010
	*PAIR1*	Rice	Essential protein for the initiation of meiotic recombination	Nonomura et al., 2004
	*OsREC8*	Rice	REC8 ortholog	Mieulet et al., 2016
	*OsOSD1*	Rice	OSD1 ortholog	Mieulet et al., 2016
Parthenogenesis	*PsASGR-BBML*	*Pennisetum squamulatum*	Regulator inducing parthenogenesis	Gualtieri et al., 2006
	*OsBBM1*	Rice	Transcription factor initiating embryo development	Khanday et al., 2019
	*ToPAR*	Dandelion	Regulator inducing parthenogenesis	Underwood et al., 2022
Genome Elimination	*CENH3*	Arabidopsis	Centromere-specific histone H3	Ravi and Chan, 2010
	*ZmMTL*/*NLD*/*ZmPLA1*	Maize	Pollen-specific phospholipase	Gilles et al., 2017; Kelliher et al., 2017; Liu et al., 2017
	*OsMATL*	Rice	MTL ortholog	Yao et al., 2018
	*ZmDMP*	Maize	Enhances and triggers haploid induction	Zhong et al., 2019
	*AtDMP8*	Arabidopsis	*ZmDMP* ortholog	Zhong et al., 2020
	*AtDMP9*	Arabidopsis	*ZmDMP* ortholog	Zhong et al., 2020

### Mimicking Apomeiosis

Bypassing or altering meiosis during embryo sac formation is a critical step for engineering apomixis in sexual plants. A mutation in Arabidopsis (*Arabidopsis thaliana*) *DYAD* (also named *SWITCH1*) can lead to apomeiosis and produce an unreduced female gamete (Ravi et al., 2008). *AMEIOTIC1* (*AM1*) in maize (*Zea mays*) is an ortholog of *DYAD*, and the *am1* mutant also displays mitotic-like division rather than a typical meiosis (Pawlowski et al., 2009). The mutation of a single gene in sexual plants can therefore give rise to functional apomeiosis. However, the *dyad* mutation is unlikely to be a good choice for engineering apomeiosis in crops because of its high rate of sterility.

**Figure 1:**
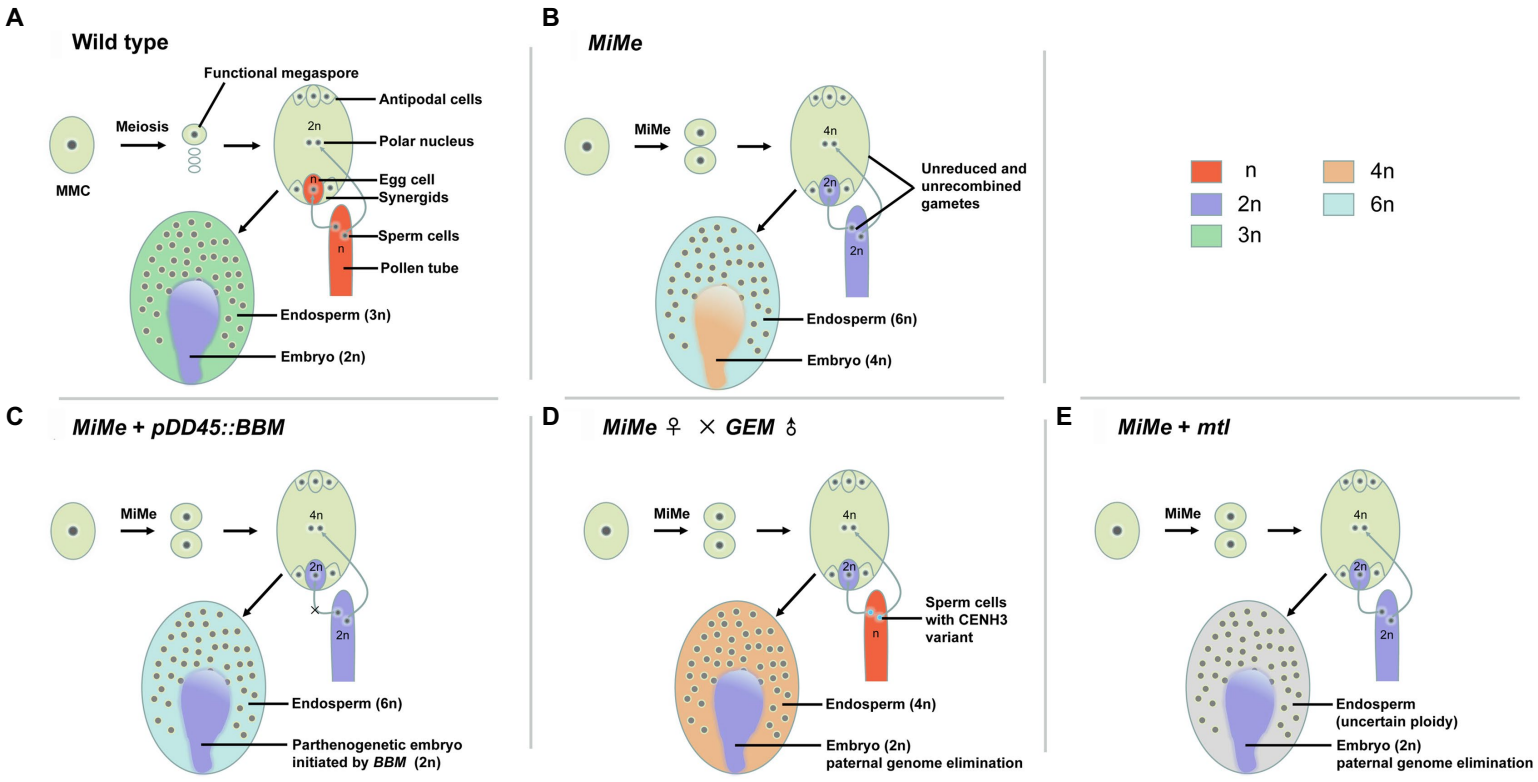
Illustration of apomixis in wild type and engineering of asexual propagation through seeds based on the *MiMe* triple mutant. **(A)** In the wild type, the megaspore mother cell (MMC) undergoes meiosis, leading to the formation of reduced and recombined haploid (n) male and female gametes. Double fertilization of the egg cell and central cell by sperm cells leads to the formation of the embryo (2n) and endosperm (3n), respectively. **(B)** In the *MiMe* triple mutant, the MMC undergoes a mitotic-like division (mitosis instead of meiosis, MiMe), leading to the formation of unrecombined and unreduced diploid (2n) gametes. The fusion of the egg cell nucleus (2n) and central polar nucleus (4n) with the sperm cell nucleus (2n) produces a tetraploid (4n) clonal embryo and hexaploid (6n) endosperm, respectively. **(C–E)** Engineering apomixis has been achieved using three schemes based on the *MiMe* triple mutant. **(C)** Combination of the *MiMe* triple mutant with the ectopic expression of *BBM* (*OsBBM1* in rice) in the egg cell triggers the formation of a parthenogenetic embryo (2n). **(D)** Crossing the male CENH3-modified genome elimination line (*GEM*) with the female *MiMe* triple mutant triggers the formation of a diploid clonal embryo (2n). **(E)** Creating the *MiMe mtl* quadruple mutant triggers the formation of a diploid clonal embryo (2n), but the ploidy of the endosperm is not known.

This challenge of high sterility was overcome by the development of an effective method called mitosis instead of meiosis (*MiMe*). The *MiMe* genotype, in which meiosis is completely replaced by a mitotic-like division without affecting subsequent sexual processes (Figures 1A,B), was first created in Arabidopsis (D’Erfurth et al., 2009). The *MiMe* genotype consists of mutations in three meiosis-specific genes: *SPORULATION 11–1* (*SPO11-1*), *REC8*, and *OMISSION OF SECOND DIVISION* (*OSD1*). In addition to these genes, *TARDY ASYNCHRONOUS MEIOSIS* (*TAM*), encoding a type-A cyclin, is required for the transition of meiosis I to meiosis II. The *tam* mutation can substitute for the *osd1* mutation to generate a *spo11-1 rec8 tam* triple mutant and create another triple mutant in which meiosis is replaced by mitosis, called *MiMe-2* (D’Erfurth et al., 2010). Similarly, the *spo11-1* mutation can be replaced by mutations in other genes, such as *PUTATIVE RECOMBINATION INITIATION DEFECT* genes (*PRD1*, *PRD2*, and *PRD3*), which are essential for the initiation of meiotic recombination to evoke the *MiMe* phenotype (Stacey et al., 2006; De Muyt et al., 2007). In addition, mutations in *HOMOLOGOUS PAIRING ABERRATION IN RICE MEIOSIS 1* (*PAIR1*), which is required for homologous chromosome pairing in rice (*Oryza sativa*) such that meiotic recombination is completely abolished in the *pair1* mutant (Nonomura et al., 2004), have been utilized in combination with mutant alleles of *Osrec8* and *Ososd1* through hybridization to transfer the *MiMe* approach to rice (Mieulet et al., 2016). With the development of targeted gene-editing technology such as clustered regularly interspaced short palindromic repeats (CRISPR)/CRISPR-associated protein 9 (Cas9), *MiMe* genotypes can be created more expediently without requiring cumbersome hybridization in the intermediate steps (Knott and Doudna, 2018). CRISPR/Cas9-mediated genome editing was used to synchronously knockout three genes in rice, *PAIR1*, *OsREC8*, and *OsOSD1* (Khanday et al., 2019) or *OsSPO11-1*, *OsREC8*, and *OsOSD1* (Xie et al., 2019), to produce *MiMe* phenotypes and achieve apomixis through additional parthenogenesis or an inducer without fertilization.

### Induced Parthenogenesis

To achieve apomixis, another pivotal step is the autonomous development of an embryo from an egg cell by parthenogenesis. *Pennisetum squamulatum*, a wild relative of pearl millet (*Pennisetum glaucum*), is a natural apomict, in which apomixis is transmitted by a large non-recombining chromosomal region named the apospory-specific genomic region (ASGR; Ozias-Akins et al., 1998). The ASGR contains multiple copies of *PsASGR-BABY BOOM-like* (*PsASGR-BBML*; Gualtieri et al., 2006), a member of the *BBML* subgroup of the *APETALA 2* (*AP2*) gene family. *PsASGR-BBML* is expressed in the ovaries, unfertilized egg cells, and developing embryos and can induce parthenogenesis. When *BBML* is expressed under the control of its own promoter in sexual pearl millet, haploid embryos are produced (Conner et al., 2015). This result highlights the significant role of *PsASGR-BBML* in parthenogenesis and might be valuable for engineering apomixis in crops. Further studies suggested that *PsASGR-BBML* can induce parthenogenesis under the control of either its own promoter or an Arabidopsis egg-cell-specific promoter [*DOWNREGULATED IN* dif1 *45* (*DD45*), At2g21740], which drives gene expression in egg cells in monocot crops, such as maize and rice (Steffen et al., 2007; Lawit et al., 2013; Ohnishi et al., 2014; Conner et al., 2017). The *ASGR-BBML* genes share high sequence similarity with *OsBBM1* (Conner et al., 2015). Unlike *PsASGR-BBML*, *OsBBM1* is expressed in sperm cells but not in egg cells (Khanday et al., 2019). The ectopic expression of *OsBBM1* in egg cells under the control of the *DD45* promoter can induce parthenogenesis in rice. Recently, the *PARTHENOGENESIS* (*PAR*) gene, which encodes a zinc finger domain protein with an EAR (ethylene-responsive element-binding factor-associated amphiphilic repression, DLNxxP) motif, was isolated from apomictic dandelion (*Taraxacum officinale*). Unlike the recessive sexual alleles, the dominant *ToPAR* allele is expressed in egg cells and has a conserved miniature inverted-repeat transposable element (MITE) transposon insertion in the promoter. The MITE-containing *ToPAR* promoter can invoke a *PAR* homolog from sexual lettuce (*Lactuca sativa*) to induce parthenogenesis (Underwood et al., 2022). The heterologous expression of the *ToPAR* gene can also induce embryo-like structures under the control of its own promoter in egg cells of sexual lettuce in the absence of fertilization. Taken together, these findings show that *PsASGR-BBML*, *OsBBM1,* and *ToPAR* are ideal genes for inducing parthenogenesis.

Somatic embryogenesis, a unique pathway for induced asexual reproduction or somatic cloning *in vitro* that bypasses the fusion of gametes, illustrates the extraordinary capacity for totipotent growth in plant cells (Su et al., 2015, 2020; Horstman et al., 2017). Somatic embryos retain the genotype of the explants and are used to asexually propagate plants to shorten the breeding cycle for species with a long reproductive cycle or highly heterozygous genomes (Park, 2002; Lelu-Walter et al., 2013; Horstman et al., 2017; Su et al., 2020). In Arabidopsis seedlings, somatic embryogenesis can be induced by the ectopic expression of certain transcription factor genes, mainly embryo-identity genes, in the absence of stress or growth regulator treatments. These include the AP2/ETHYLENE RESPONSE FACTOR (ERF) transcription factor gene *BBM* (Boutilier et al., 2002) and most of the genes encoding the network of LAFL proteins, including the HAP3 family of CCAAT-binding factors composed of LEAFY COTYLEDON 1 (LEC1) and LEC1-LIKE (L1L), and a subgroup of the plant-specific B3 domain protein family including LEAFY COTYLEDON 2 (LEC2), FUSCA3 (FUS3), and ABSCISIC ACID INSENSITIVE 3 (ABI3; Giraudat et al., 1992; Lotan et al., 1998; Luerßen et al., 1998; Stone et al., 2001; Kwong et al., 2003; Jia et al., 2013; Tang et al., 2020). As mentioned earlier, *PsASGR-BBML* and *OsBBM1* can induce parthenogenesis, whereby embryos develop from female gametophytic cells (egg cells). *OsBBM1* is expressed in sperm cells (Khanday et al., 2019) and functions as paternal factors that can trigger embryogenesis, perhaps by activating silent maternal transcripts. To date, none of the LAFL genes have been reported to induce parthenogenesis. Whether the other proteins can mimic BBM and confer totipotency to unfertilized egg cells to achieve parthenogenesis remains unknown. Those genes whose overexpression can induce somatic embryogenesis as *BBM* are considered as candidate genes to induce parthenogenesis.

### Genome Elimination

The aim of engineering apomixis is to retain desirable traits harbored by the uniparental genotype. Genome elimination from a diploid zygote postfertilization may be another means to engineer apomixis. Eliminating one set of maternal or paternal chromosomes after fertilization can therefore achieve haploid induction. In plants, centromere-specific histone H3 (CENH3) can be used to identify the centromeres, chromosomal regions where the spindle microtubules are anchored to mediate chromosome segregation during cell division (Henikoff and Dalal, 2005). *cenh3-1* is an embryo-lethal null mutation in Arabidopsis but can be fully complemented by the expression of a transgene encoding a green fluorescent protein (GFP)-tagged CENH3 (GFP-CENH3) fusion protein. In addition, the embryo lethality of *cenh3-1* can be rescued using GFP-tailswap, in which the hypervariable N-terminal tail domain of CENH3 is replaced with the tail domain of conventional histone H3. When plants rescued by *GFP-tailswap* or *GFP-CENH3* are used as male or female parents in a cross with the wild-type genotype containing unaltered CENH3, the genomes of the transgenic plants carrying mutant *cenh3* are eliminated, and haploid offspring containing the genome of only one parent are generated (Ravi and Chan, 2010). The plants rescued with *GFP-tailswap* can induce haploids effectively but are largely male-sterile due to the defect in meiosis. Conversely, the *GFP-CENH3* transgene mostly rescues fertility, but the frequency of haploid induction is much lower. Subsequently, a CENH3-modified genome elimination (*GEM*) line was produced, in which *GFP-CENH3* and *GFP-tailswap* are co-expressed to rescue the *cenh3-1* mutant. The *GEM* plants are fully fertile and can be used as either male or female parents in crosses. *GEM* plants also effectively lead to genome elimination when crossed to plants containing wild-type *CENH3*. Finally, clonal seeds (doubled haploids) can be generated by crossing the *GEM* line to male or female *MiMe* plants in Arabidopsis (Marimuthu et al., 2011).

Haploid induction can also occur spontaneously in nature, albeit infrequently (Gilles et al., 2017), and is routinely used in maize breeding. Recently, the molecular basis of haploid induction in maize was uncovered through fine-mapping and genome sequencing. A frameshift mutation in *MATRILINEAL* (*MTL or MATL*), also known as *PHOSPHOLIPASE A1* and *NOT LIKE DAD* (*NLD*), whose wild-type allele encodes a pollen-specific phospholipase, triggers maternal haploid induction in maize (Gilles et al., 2017; Kelliher et al., 2017; Liu et al., 2017). *OsMATL*, a putative *ZmMTL* ortholog, is responsible for maternal haploid induction in rice. A knockout mutation in *OsMATL* leads to a 2–6% haploid induction rate when these plants are self-pollinated or outcrossed as the male parent (Yao et al., 2018). The underlying mechanism of haploid induction caused by the mutation of *MTL* remains unclear but may involve the selective elimination of uniparental chromosomes or the continuous chromosome fragmentation that takes place after meiosis in the gametophyte (Zhao et al., 2013; Li et al., 2017). In addition, a mutation in *DOMAIN MEMBRANE PROTEIN* (*DMP*), another gene expressed specifically in pollen, can lead to independent haploid induction and significantly improve the haploid induction rate in the presence of *mtl* in maize (Zhong et al., 2019). *MTL* is conserved only in monocots, while *DMP*-*like* genes exist in both eudicots and monocots and function similarly. Loss-of-function mutations in Arabidopsis *DMP8* and *DMP9* trigger haploid induction (Zhong et al., 2020). Induction of haploid plants from the genetic disruption of *MTL* or *DMP* is another form of paternal genome elimination. It would be possible to produce diploid clonal seeds *via* the simultaneous engineering of *MiMe* with loss-of-function in *MTL* or *DMP*.

### Composite Methods for Engineering Apomixis

Using the methods mentioned above, the introduction of apomixis into sexual plants has become a reality by combining the *MiMe* system with other genetic pathways that trigger the formation of uniparental embryos. Three composite methods (Figures 1C–E) are currently in use for triggering apomixis in sexual plants. First, asexual propagation through engineered apomixis can be achieved by combining the *MiMe* triple mutant with the ectopic expression of rice *OsBBM1* in egg cells (Khanday et al., 2019). Using this approach, offspring retaining the same ploidy as the mother plants can be obtained at frequencies of 11–29%. This apomixis is heritable through multiple generations of clones, as demonstrated by whole-genome sequencing. Second, diploid clonal progenies can be generated by crossing the *GEM* line with male or female *MiMe* triple mutant plants in Arabidopsis, reaching frequencies of 24% (*MiMe* as female) or 42% (*MiMe* as male). The diploid clonal progeny retains the heterozygosity of the *MiMe* parent, providing evidence of clonal reproduction (Marimuthu et al., 2011). Third, engineered apomixis can be introduced into rice by creating quadruple mutants using the CRISPR/Cas9 system: *Osrec8 pair1 Ososd1 Osmatl* (named *Fix*, for *Fixation of hybrids*; Wang et al., 2019) and *Osspo11-1 Osrec8 Ososd1 Osmatl* (named *AOP*, for *Apomictic Offspring Producer*; Xie et al., 2019), resulting in plants that propagate clonally through their seeds. In this third approach, unreduced clonal female gametes develop into embryos through haploid induction in the absence of the paternal genome. However, the *Fix* or *AOP* quadruple mutants display reduced fertility and a low haploid induction rate caused by the mutation in *OsMATL*; for example, the haploid induction rate in *Fix* is 4.7–9.5% (Wang et al., 2019; Xie et al., 2019).

## Concluding Remarks and Perspective

Apomixis in crops will not only facilitate the maintenance of elite hybrid genotypes but also shorten the breeding cycle of heterozygous plants to simplify hybrid production strategies. Recent publications have illustrated the diverse components of apomixis (Fayos et al., 2019; Kaushal et al., 2019; Vijverberg et al., 2019; Ozias-Akins and Conner, 2020). In this review, we systematically summarized the types of apomixis that spontaneously occur in nature and focused on inducing apomixis. For engineering apomixis, there are three schemes widely used as: combining *MiMe* with the ectopic expression of *OsBBM1*; crossing the CENH3-modified *GEM* line with *MiMe*; and creating quadruple mutants containing *MiMe* and *mtl*. The *MiMe* genotype is an effective system for replacing meiosis by mitosis, although it requires the simultaneous genetic inactivation of three genes. This obstacle can be overcome using CRISPR/Cas9 systems by mutating multiple genes without requiring cumbersome hybridization in the intermediate steps. For parthenogenesis, *PsASGR-BBML* has been verified as the parthenogenesis-inducing gene, following a demonstration of its functionality in sexual pearl millet, maize, and rice. The ectopic expression of *OsBBM1* in egg cells can also induce parthenogenesis in rice. Driving the expression of a *PAR* gene in egg cells of dandelion can bring about parthenogenesis. Whether other proteins can mimic BBM or PAR to achieve parthenogenesis remains unknown. Identifying candidate genes for parthenogenesis, and then transferring this knowledge to crop species for its application to plant breeding, is the goal.

## Author Contributions

PPY and YHS designed and wrote the manuscript. LPT wrote some parts of the manuscript. XSZ gave valuable comments on the review. All authors have read and agreed to the published version of the manuscript.

## Funding

This work was funded by the National Natural Science Foundation of China (31730008, 31872669, and 32070199) and the Program for Scientific Research Innovation Team of Young Scholar in Colleges and Universities of Shandong Province (2019KJE011).

## Conflict of Interest

The authors declare that the research was conducted in the absence of any commercial or financial relationships that could be construed as a potential conflict of interest.

## Publisher’s Note

All claims expressed in this article are solely those of the authors and do not necessarily represent those of their affiliated organizations, or those of the publisher, the editors and the reviewers. Any product that may be evaluated in this article, or claim that may be made by its manufacturer, is not guaranteed or endorsed by the publisher.
